# Personality Functioning in Inpatients With Eating Disorders: Association With Symptom Severity and Treatment Outcome

**DOI:** 10.1002/erv.3183

**Published:** 2025-02-17

**Authors:** Sophia Heinzmann, Sonja Etzler, Armin Hartmann, Eva M. Klein, Stephan Herpertz, Magdalena Pape, Stanislav Heinzmann, Stephan Doering, Tobias Hofmann, Matthias Rose, Katrin Imbierowicz, Franziska Geiser, Antonie Bierling, Kerstin Weidner, Jörg Rademacher, Silke Michalek, Eva Morawa, Yesim Erim, Eva‐Maria Skoda, Martin Teufel, Eva Milena Johanne Peters, Johannes Kruse, Dirk von Boetticher, Christoph Herrmann‐Lingen, Mariel Noehre, Martina de Zwaan, Ulrike Dinger, Hans‐Christoph Friederich, Alexander Niecke, Christian Albus, Rüdiger Zwerenz, Manfred Beutel, Casper Roenneberg, Peter Henningsen, Barbara Stein, Christiane Waller, Karsten Hake, Carsten Spitzer, Andreas Stengel, Stephan Zipfel, Katja Weimer, Harald Gündel, Henrik Kessler, Derek Spieler, Claas Lahmann, Almut Zeeck

**Affiliations:** ^1^ Faculty of Medicine Department of Psychosomatic Medicine and Psychotherapy Center for Mental Health University of Freiburg Freiburg Germany; ^2^ Protestant University of Applied Sciences Freiburg Freiburg Germany; ^3^ Institute of Psychology Goethe‐University Frankfurt Main Frankfurt am Main Germany; ^4^ Department of Psychosomatic Medicine and Psychotherapy LWL‐University Hospital Ruhr‐University Bochum Bochum Germany; ^5^ Department of Clinical Psychology and Psychotherapy University of Bamberg Bamberg Germany; ^6^ Department of Psychoanalysis and Psychotherapy Medical University of Vienna Vienna Austria; ^7^ Comprehensive Clinical Center for Neurosciences and Mental Health (C3NMH) Medical University of Vienna Vienna Austria; ^8^ Department of Psychosomatic Medicine Charité Center for Internal Medicine and Dermatology Charité‐Universitätsmedizin Corporate Member of Freie Universität Berlin and Humboldt‐Universität zu Berlin Berlin Germany; ^9^ German Center for Mental Health (DZPG) Partner Site Berlin/Potsdam Berlin Germany; ^10^ Center for Patient‐Centered Outcomes Research (CPCOR) Charité ‐ Universitätsmedizin Berlin Berlin Germany; ^11^ Department of Psychosomatic Medicine and Psychotherapy DRK Kliniken Berlin Wiegmann Klinik Berlin Germany; ^12^ Department of Psychosomatic Medicine and Psychotherapy University Hospital University of Bonn Bonn Germany; ^13^ Faculty of Medicine and University Hospital Carl Gustav Carus Department of Psychotherapy and Psychosomatic Medicine TUD University of Technology Dresden Dresden Germany; ^14^ Department of Clinical Psychology Friedrich‐Schiller University Jena Germany; ^15^ Department of Psychosomatic Medicine and Psychotherapy LVR‐University Hospital Heinrich Heine University Düsseldorf Düsseldorf Germany; ^16^ Department of Psychosomatic Medicine and Psychotherapy University Hospital of Erlangen Friedrich‐Alexander University Erlangen‐Nuremberg Erlangen Germany; ^17^ Clinic of Psychosomatic Medicine and Psychotherapy LVR‐University Hospital University of Duisburg‐Essen Essen Germany; ^18^ Department of Psychosomatic Medicine and Psychotherapy Germany and Department of Psychosomatic Medicine and Psychotherapy Justus‐Liebig University of Giessen Philipps‐University of Marburg Marburg Germany; ^19^ Department of Psychosomatic Medicine and Psychotherapy University Medical Centre Göttingen Göttingen Germany; ^20^ Department of Psychosomatic Medicine and Psychotherapy Hannover Medical School Hannover Germany; ^21^ Department of General Internal Medicine and Psychosomatics University Hospital Heidelberg University Heidelberg Germany; ^22^ Faculty of Medicine and University Hospital Cologne Department of Psychosomatic Medicine and Psychotherapy University of Cologne Cologne Germany; ^23^ Department of Psychosomatic Medicine and Psychotherapy University Medical Center of the Johannes Gutenberg University Mainz Mainz Germany; ^24^ Department of Psychosomatic Medicine and Psychotherapy University Hospital Technical University of Munich Munich Germany; ^25^ Department of Psychosomatic Medicine and Psychotherapy Nuremberg General Hospital Paracelsus Medical University Nuremberg Germany; ^26^ Department of Psychosomatic Medicine and Psychotherapy University Medical Center Rostock Rostock Germany; ^27^ Clinic for Psychosomatic Medicine and Psychotherapy Klinikum Stuttgart Stuttgart Germany; ^28^ Internal Medicine VI Psychosomatic Medicine and Psychotherapy University Hospital Tübingen Tübingen Germany; ^29^ German Centre for Mental Health (DZPG) University of Tuebingen Tuebingen Germany; ^30^ Department of Psychosomatic Medicine and Psychotherapy Ulm University Medical Center Ulm Germany; ^31^ Department of Psychosomatic Medicine and Psychotherapy Campus Fulda University of Marburg Marburg Germany

**Keywords:** eating disorder, inpatient, latent change score model, personality functioning, psychotherapy

## Abstract

**Objective:**

Impairment in personality functioning (PF) has been linked to a number of mental disorders, including eating disorders (EDs). However, the precise relationship between PF and symptom severity, as well as the potential impact on outcome, remains unclear. The study aimed to analyse the association of PF and its change with severity of ED symptomatology as well as outcome of hospital treatment.

**Method:**

The sample consisted of 397 patients with EDs, treated in 19 university hospitals for Psychosomatic Medicine and Psychotherapy in Germany between 1/2019 and 12/2020. PF was measured with the Structure Questionnaire of the Operationalised Psychodynamic Diagnosis (OPD‐SQ, short version), eating psychopathology with the ED examination questionnaire (EDE‐Q). Outcome was defined as a change in the EDE‐Q total score. We used Latent Change Score Modelling to analyse changes in ED pathology during treatment and a 1‐year follow‐up period.

**Results:**

A higher level of impairment in PF at admission correlated with more eating psychopathology and a less favourable outcome. Additionally, greater improvement in PF correlated with greater improvements in ED symptomatology at discharge.

**Conclusion:**

Impairment in PF needs to be part of diagnostic assessments and should be considered an important treatment target for psychotherapeutic interventions.

**Trial Registration:**

The MEPP study was registered in the German Clinical Trials Register (DRKS, www.drks.de; ID: DRKS00016412)

**Summary**

- Impairment in personality functioning is associated with more severe ED psychopathology.
- Patients with more impairment in personality functioning show a less favourable course in hospital treatment for EDs.
- Improvement in personality functioning during treatment is correlated with a more favourable course of the ED.

## 1 Introduction

Eating disorders (EDs) such as anorexia nervosa (AN), bulimia nervosa (BN), binge eating disorder (BED) or atypical forms often lead to severe restrictions in patients' quality of life, serious physical consequences (Jenkins et al. 2011; Treasure, Duarte, and Schmidt 2020) as well as high health care costs (Agh et al. 2016). Overall, remission rates across diagnostic categories are unsatisfactory with under 50% (Solmi et al. 2024). In more severe cases and most often in AN, hospital treatment is warranted (AWMF 2020; NICE 2020). Indication criteria are oriented on the physical situation, severity of eating pathology and severity of overall mental disturbance including suicidal ideation and comorbidity.

EDs show high rates of comorbidity with several other mental disorders including personality disorders (Martinussen et al. 2017). Comparing different ED diagnoses, the mean proportion for any personality disorder was similar in AN (0.49) and BN (0.54) and somewhat lower in BED (0.39) and EDs not otherwise specified (EDNOS, 0.29) (Friborg et al. 2014; Martinussen et al. 2017). A recent systematic review by Simpson et al. (2022) on the impact of personality disorders on treatment outcome in EDs found that comorbid personality disorders and personality traits were associated with more problematic courses and higher attrition rates in AN and with less change in overall psychological impairment, but not with less change in bulimic symptoms in BN. Despite a limited number of studies, the authors suggest that tailoring interventions to address personality disorder symptoms additionally to ED symptoms may enhance treatment outcomes (Simpson et al. 2022).

The review by Simpson et al. is based on studies using a categorical definition of personality disorders (Simpson et al. 2022). This is a relevant limitation, as the conceptualisation of personality pathology has changed after intense debates in recent decades (Kass et al. 1985; Oldham and Skodol 2000; Sharp and Wall 2021, S. 2021; Zimmermann et al. 2019). In the new versions of the classification systems, a categorical definition is abandoned or at least supplemented by the suggestion of a dimensional model (American Psychiatric Association [APA], 2013; Tyrer et al. 2019). This model defines personality disorders through significant impairment in personality functioning (Criterion A) and the presence of maladaptive personality traits (Criterion B). Personality functioning (PF) refers to the dimensions of self and interpersonal functioning (Pincus, Cain, and Halberstadt 2020). In this approach, a personality disorder is diagnosed only when there is a significant impairment in overall personality functioning (Criterion A) along with the presence of maladaptive personality traits (Criterion B), making personality functioning a key part of a personality disorder.

A second key aspect of this new model of personality disorders is the dimensional conceptualisation of both PF and maladaptive personality traits. It is suggested that a dimensional approach has greater clinical utility by allowing a more effective targeting of interventions (Bach et al. 2020). Furthermore, dimensional models exhibit higher reliability (e.g., across raters and over time), take into account the distributional properties of personality disorders, and consider the observable symptom heterogeneity of different personality disorders (Trull and Durrett 2005). In terms of its prognostic relevance, moderate or severe impairment in PF was found to be associated with an increased risk of negative treatment outcomes (Bach et al. 2020). While personality functioning serves as primary key criterion for personality disorders, research suggests that it might also serve as a transdiagnostic construct relevant to mental distress and disorders such as depression, anxiety (Freier et al. 2022), and eating psychopathology (Klein et al. 2022).

The assessment of PF has a long tradition in psychodynamic theory and diagnostics, for example in ‘object relations theory’ (Clarkin, Caligor, and Sowislo 2020). It is postulated that relationship experiences in early childhood significantly influence the development of mental abilities such as impulse and affect regulation or the ability to engage in close relationships. These experiences interact with biological and social influences or temperamental traits (Caligor et al. 2018; Kernberg and Caligor 2005). One instrument, which is based on the psychodynamic model and was developed for the assessment of PF (respectively the assessment of ‘personality structure’) is the structure axis of the Operationalised Psychodynamic Diagnostic System (OPD; Arbeitskreis OPD 2009; Zimmermann et al. 2012). Its conceptualisation has many similarities with the definition of PF in the alternative model of personality disorders in the DSM‐5 and the dimensional model of personality disorders in the ICD‐11 (criterion A; (American Psychiatric Association [APA], 2013; Zimmermann et al. 2012 2015). Therefore, the structure axis in OPD interviews and self‐report forms such as the OPD‐structure‐questionnaire assesses PF as intended by DSM‐5 and ICD‐11 (Zimmermann et al. 2020).

Previous research on PF in patients with an ED is limited, but may be of high relevance for adapted treatment strategies, especially in the group of patients requiring inpatient or day hospital treatment. Rohde et al. (2019 2023) compared hospitalised patients with the restrictive subtype of AN (AN‐R), patients with the binge‐purging subtype of AN (AN‐BP) and patients with BN according to PF, using the OPD structure questionnaire OPD‐SQ in a pilot study (*N* = 60) and a main study (*N* = 110). The focus of the studies was on group differences. The AN‐R group showed less impairment in PF compared to patients with AN‐BP and BN. The groups also showed differences in various areas of PF, which may be relevant for psychotherapeutic treatment, such as tolerance of affect. Severity of eating pathology showed a statistically significant, moderate positive correlation with impairment in PF (Rohde et al. 2023). Another study explored the relationship between PF and several personality disorder‐clusters in relation to ED pathology and treatment outcome in 107 adult hospitalised women with ED problems (Muzi et al. 2021a). The level of PF (including aspects such as empathy, affect regulation, capacity to get involved in intimate relationships and reflective capacities) predicted treatment outcome (severity of ED symptoms): Patients with higher levels of PF had better outcomes at discharge compared with patients with lower levels of PF. Furthermore, higher levels of PF mitigated the effect of baseline symptom severity on outcome (Muzi et al. 2021a).

In sum, these findings need to be replicated in studies using a dimensional definition of personality disorder and which are conducted not only in a single centre, but include a more representative sample of patients and a follow‐up period. Before identifying the areas of impaired PF in EDs and their relationship to ED symptoms in more detail, the relationship between the level of impairment in PF in general and severity of ED psychopathology as well as treatment outcome should be studied further.

Against this background, the aim of this study was to analyse the relationship between PF and severity of ED psychopathology as well as the influence of baseline PF on the outcome of hospital treatment in patients with EDs consecutively admitted for hospital treatment in Germany university hospitals over a period of 2 years. Additionally, the aim was to explore changes in PF over the course of treatment in this group and whether these changes were related to outcome. We hypothesised that a stronger impairment in PF will be associated (1) with a higher level of ED psychopathology and (2) with a poorer treatment outcome; we further assumed (3) that improvement in PF will be associated with a better outcome.

## 2 Method

### 2.1 Study Design

The study is a secondary data analysis and uses data from the Multicenter Effectiveness Study of Inpatient and Day Hospital Treatment in Departments of Psychosomatic Medicine and Psychotherapy (MEPP) in Germany (Doering, Herpertz, Hofmann, et al. 2023; Doering, Herpertz, Pape, et al. 2023). In the MEPP‐study, 2094 consecutively admitted patients with various diagnoses for which inpatient or day‐hospital treatment was indicated and who were treated in 19 participating university hospitals between 1/2019 and 12/2020 were included.

Time points of assessment were admission (T0), discharge (T1) and a follow‐up assessment 1 year after discharge (T2). Assessments entailed interviews by trained clinicians at admission and self‐report instruments at admission, discharge and follow‐up. The structured interviews at admission were used to diagnose mental disorders including PDs according to ICD‐10. The questionnaires were administered to record the symptoms of various disorders including EDs and PF at baseline (admission) and their change from T0 to T1 and T2.

The MEPP study was registered in the German Clinical Trials Register (DRKS, www.drks.de; ID: DRKS00016412) (Doering, Herpertz, Pape, et al. 2023).

### 2.2 Sample

For the present study, the subgroup of patients with an ED (*N* = 397) was examined. The sample was 89% female with a mean age of 33.4 years (SD = 13.6). It included all patients who had been diagnosed in a standardized clinical interview with an ED according to ICD‐10, that is patients with a diagnosis of (atypical) anorexia nervosa (F50.0, F50.1), (atypical) bulimia nervosa (F50.2, F50.3) and with an otherwise specified or not otherwise specified ED (F50.8, F50.9). *N* = 15 Patients with the diagnoses ‘overeating associated with other psychological disturbances’ (F50.4) and ‘vomiting associated with other psychological disturbances’ (F50.5) were excluded from the total sample, as ED pathology in these groups is part of another mental disorder. We also excluded *n* = 18 patients who spent less than 14 days in treatment, as these patients did not receive sufficient treatment to assume a significant treatment effect. These patients usually leave treatment early due to low motivation, are admitted for diagnostic reasons only, or are discharged because the treatment programme has proved not to be suitable. For further characteristics of the sample see Table 1.

### 2.3 Measures

Trained clinical raters provided ICD‐10 and DSM‐IV diagnoses based on two structured clinical interviews at baseline: The Diagnostic Interview for Mental Disorders (Mini‐DIPS; Margraf et al. 2017; Margraf and Cwik 2023), a German‐language interview for recording all mental disorders, comparable to the Structured Interview for DSM‐IV (SCID‐I), which enables conversion to ICD‐10 diagnoses and the German version of the Structured Interview for DSM‐IV Axis II (SCID‐II) to assess personality disorders (Fydrich et al. 1997). Up to 10 diagnoses could be recorded for each patient.

Personality functioning (as a general factor for overall impairment in psychic capacities) was measured with the total score of the OPD‐SQS (Ehrenthal et al. 2015). Based on the OPD structure axis, the OPD‐SQS is the short, 12‐item version of the OPD ‐ Structure Questionnaire (OPD‐SQ) that consists of 95 items. The items are rated on a five‐point scale ranging from ‘fully disagree’ to ‘fully agree’. There are items that refer to mental abilities related to the self (e.g. ‘I sometimes feel like a stranger to myself.’) and others that ask about interpersonal functioning (related to a relationship model: e.g. ‘It can be dangerous to let others get too close to you.’ or on getting in contact with others: e.g. ‘I find it difficult to make others understand me.’). Ehrenthal et al. (2015 2023) reported good internal consistency of the OPD‐SQS scale (Cronbach's *α* = 0.88 and McDonalds *ω* = 0.93). They also found that the OPD‐SQS scores differed significantly between inpatients, outpatients and those not in treatment, and between patients with and without a personality disorder diagnosis, confirming the validity of the OPD‐SQS (Ehrenthal et al. 2015). In a sample of 565 outpatients and 670 inpatients, Obbarius et al. (2019) found significant correlations of OPD‐SQS scores with SCID‐II self‐report questionnaire scores for most DSM‐IV PDs and with the dimensional scores of SCID‐II interview ratings for five of the 12 DSM‐IV PDs (Obbarius et al. 2019). Regarding the dimensional models of PD, Zimmermann et al. (2020) found that the OPD‐SQS as well as five other self‐report measures for PF loaded strongly on a general factor for PF, showing that these instruments are appropriate measures for the latent continuum of PF (Zimmermann et al. 2020). The OPD‐SQS was also found to be suitable for measuring changes in PF as a result of treatment in psychotherapy inpatients (Flemming et al. 2022; Lübke et al. 2021). In the sample of the current study, the OPD‐SQS demonstrated excellent internal consistency, with a Cronbach's *α* = 0.88 and McDonald's *ω* total = 0.90, indicating high reliability of the instrument.

Eating psychopathology was measured with the German version of the Eating Disorder Examination Questionnaire (EDE‐Q; Hilbert and Tuschen‐Caffier 2016). The EDE‐Q is a widely used self‐report measure for assessing the type and severity of ED symptoms. It is an adaptation of the Eating Disorder Examination interview and consists of 28 items that evaluate key attitudes and behaviours related to EDs over the past 28 days. The items are rated on a seven‐point scale ranging from ‘no days’ to ‘every day’ for frequency questions and from ‘not at all’ to ‘markedly’ for intensity questions. The EDE‐Q generates four subscale scores—Restraint, Eating Concern, Shape Concern, and Weight Concern—along with a global score that reflects overall symptom severity. There are additional items for height, weight and current amenorrhoea that were not used in this study. The EDE‐Q shows good to very good reliability (subscales: 0.85 ≤ Cronbach's *α* ≤ 0.93; total score: Cronbach's *α* = 0.97), the subscales remain stable over a 3‐month period, with moderate stability of core behavioural items (Hilbert and Tuschen‐Caffier 2016). Convergent and criterion validity have been established in various studies (Hilbert and Tuschen‐Caffier 2016). In the sample of the current study, the EDE‐Q demonstrated excellent internal consistency, with a standardized Cronbach's *α* = 0.93 and McDonald's *ω* total = 0.95, indicating high reliability.

### 2.4 Definition of Outcome

Outcome was defined as a change of the total score of the EDE‐Q (eating disorder pathology) from T0 to T1 (over the course of treatment: admission to discharge) and from T1 to T2 (follow‐up period: discharge to 1 year after discharge).

### 2.5 Intervention

Patients received the usual, multimodal inpatient and/or day hospital treatment in one of 19 university hospitals for psychosomatic medicine. Treatment in all hospitals followed German health care standards (Doering, Herpertz, Pape, et al. 2023). The treatment of patients with an ED is additionally oriented on the German treatment guidelines for the diagnosis and treatment of EDs (Herpertz et al. 2011). It was multidisciplinary, bio‐psycho‐socially oriented and integrative, encompassing elements of psychodynamic, cognitive behavioural and systemic therapy. Each patient received at least three sessions per week of individual and/or group psychotherapy, and additional treatments, for example psychopharmacological therapy, creative, body‐oriented or mindfulness‐based therapies, psychoeducation, support by the nursing staff (including nutrition management in AN) or social work assistance (Doering, Herpertz, Pape, et al. 2023). ED treatment has a focus on eating pathology, as well as on psychological problem areas (e.g. self‐esteem, affect regulation).

Indications for inpatient treatment in patients with EDs include insufficient outpatient outcomes and/or the need for a more intensive treatment programme, for example in case of chronicity, severe co‐morbidity, somatic risk (e.g. BMI < 15 kg/m^2^, electrolyte disturbances) and the need for continuous meal supervision. Exclusion criteria for psychosomatic treatment programs are psychosis, substance dependency other than nicotine, bipolar disorder, organic brain disease, dementia, severe somatic complications or acute suicidal ideation.

### 2.6 Statistical Analyses

Means, standard deviations and frequencies were computed for the description of the sample. Mann‐Whitney‐U‐Tests were calculated to determine if there were differences in ED symptom severity and PF between patients who left treatment after less than 14 days and those who stayed longer.

To examine our hypotheses, we conducted two Latent State Change Score Models (LCSM). Latent Change Modelling can be used to analyse and quantify changes in latent variables over time. It extends traditional structural equation modelling (SEM) by incorporating a dynamic component to examine how and why variables change. This allows the assessment of interindividual differences in intraindividual change trajectories and the exploration of factors that contribute to variability in these trajectories (Geiser 2021, 156). LCSM allowed to model changes in ED pathology without assuming a certain growth shape or curve, using Latent Change Scores instead; this approach is appropriate for our data with three time points of data collection.

Both LCSM were estimated using maximum likelihood estimation with the *Nonlinear Minimisation with Bounds* (NLMINB) optimization method, which accounts for missing data under the assumption of *Missing At Random* (MAR). In both models, 394 of 397 observations were used, there were nine and ten patterns of missing data in Model 1 and Model 2, respectively. Full Information Maximum Likelihood (FIML) estimation was used to handle missing data. FIML estimation of model parameters maximises a likelihood function that is based on complete and incomplete cases (Enders and Bandalos 2001). The FIML algorithm uses all available data for parameter estimation, that is a case is used for the estimation of all parameters for which it has complete data.

Model fit for LCSMis usually examined with the Root Mean Square Error of Approximation (RMSEA), the Standardized Root Mean Square Residual (SRMR), the Comparative Fit Index (CFI) and the Tucker‐Lewis Index (TLI) (Hu & Bentler PM 1999; Schermelleh‐Engel, Moosbrugger, and Müller 2003). Common cut‐offs for a good to acceptable fit are ≥ 0.95 for CFI and TLI, ≤ 0.08 for RMSEA and ≤ 0.10 for SRMR (Hu & Bentler PM 1999; Schermelleh‐Engel, Moosbrugger, and Müller 2003). However, we calculated a saturated, fully identified model. The aforementioned indices could thus not be interpreted meaningfully and are therefore not reported.

All analyses were conducted using the lavaan‐package (Rosseel 2012) in the statistics software R (R Core Team 2024).

## 3 Results

### 3.1 Descriptive Statistics

Descriptive statistics of the current study are reported in Table 1, correlation coefficients for the model based variables in Table 2. To analyse systematic differences between the sample and patients being treated less than 14 days, Mann‐Whitney‐U‐Test was calculated. It showed a significant difference in EDE‐Q values at admission, with patients who left treatment after less than 14 days showing a lower average symptom severity (*Median* = 2.00) than patients who stayed longer (*Median* = 3.57), *W* = 1550.5, *z* = 2.21, *p* = 0.03. Patients who left treatment after less than 14 days also showed lower average impairment in PF (*Median* = 2.00) compared to patients who stayed longer (*Median* = 2.42), *W* = 1656, *z* = 2.06, *p* = 0.04. Treatment duration varied between 14 and 238 days (*M* = 58.74 days, *SD* = 27.48).

**TABLE 1:** Sample description.

Variable	M (SD)/N (%)
Age [years]	33.36 (12.55)
Gender	88.92% female
Marital status	
Single/never married	173 (43.57%)
Married or living as such	160 (40.30%)
Separated/divorced/widowed	43 (10.83%)
NA	21 (5.28%)
	
Education	
> 10 years	286 (72.04%)
Occupation	
Student	101 (25.44%)
Unemployed	78 (19.65%)
Employed	168 (42.32%)
Retired	27 (6.80%)
NA	23 (5.79%)
EDE‐Q	
Admission (T0)	3.55 (1.45)
Discharge (T1)	2.78 (1.43
1‐year follow‐up (T2)	2.91 (1.54)
OPD‐SQS	
Admission (T0)	2.34 (0.82)
Discharge (T1)	2.09 (0.91)
1‐year follow‐up (T2)	2.04 (0.95)
ED diagnosis	
Anorexia nervosa	86 (21.66%)
Atypical anorexia nervosa	56 (14.11%)
Bulimia nervosa	100 (25.19%)
Atypical bulimia nervosa	14 (3.53%)
Other and unspecified ED	141 (35.52%)
PD diagnosis	
Cluster A	6 (1.51%)
Cluster B	50 (12.59%)
Cluster C	108 (27.20%)
Not otherwise specified	63 (15.87%)

Abbreviations: ED = eating disorder; EDE‐Q = Eating Disorder Examination Questionnaire; M = mean; N = number; NA = missing data; OPD‐SQS = OPD Structure Questionnaire short version; PD = personality disorder; SD = standard deviation.

**TABLE 2:** Correlation matrix.

	EDE‐Q mean T0	EDE‐Q mean T1	EDE‐Q mean T2	OPD‐SQS mean T0	OPD‐SQS change
EDE‐Q mean T0	1.00				
EDE‐Q mean T1	0.64	1.00			
EDE‐Q mean T2	0.54	0.67	1.00		
OPD‐SQS mean T0	0.38	0.44	0.34	1.00	
OPD‐SQS change	−0.05	0.19	0.21	−0.21	1.00

*Note:* T0 = admission; T1 = discharge; T2 = 1 year follow‐up.

Abbreviations: EDE‐Q = Eating Disorder Examination Questionnaire; OPD‐SQS = OPD Structure Questionnaire short verison.

A further look at the clinical characteristics of the patients indicated that only four individuals had more than one ED diagnosis. However, the majority of patients (*N* = 370; 93.2%) had at least one comorbid Axis I‐disorder. The most common comorbidities were somatoform disorders (*N* = 75), sleep disorders (*N* = 75), depressive disorders and dysthymia (*N* = 61), anxiety and panic disorders (*N* = 52) and PTSD and adjustment disorders (*N* = 49). *N* = 227 participants (59.9%) were diagnosed with at least one PD and 114 patients of these were diagnosed with more than one personality disorder. Patients diagnosed with at least one personality disorder showed significantly more impairment in PF (*Median* = 2.67) than those without any personality disorder diagnoses (*Median* = 2.04), Mann‐Whitney *U*‐Test, *W* = 10,045, *z* = 7.71, *p* < 0.001.

A correlation matrix (EDE‐Q and OPD‐SQS) among PF and ED psychopathology as utilised in the LCSM can be found in Table 2.

#### 3.1.1 Model Description

For both models, latent variables representing the severity of ED psychopathology were estimated from participants' mean overall‐scores on the EDE‐Q at admission (T0), discharge (T1) and 1‐year follow‐up (T2). The latent change in ED psychopathology is expressed in a latent change score variable or factor (LCF) for each time segment. The LCFs reflect interindividual differences in intraindividual changes between T0 and T1 (LCF1) and T1 and T2 (LCF2), respectively (Geiser 2021, 157). The change score variables are regressed on the preceding latent state variable (Geiser 2021, 158), that is the change in ED pathology during treatment (LCF1) is regressed on ED symptom severity at admission and the change in ED pathology during the follow‐up period (LCF2) is regressed on ED symptom severity at discharge. All intercepts were fixed to 0 and all loadings for the latent variables were fixed to 1, the error variances were set equal across time for the same indicator. In total, there were 16 estimated parameters and two equality constraints in both models. Both models have 0 degrees of freedom, that is we estimated a saturated model.

#### 3.1.2 Model 1

**FIGURE 1:**
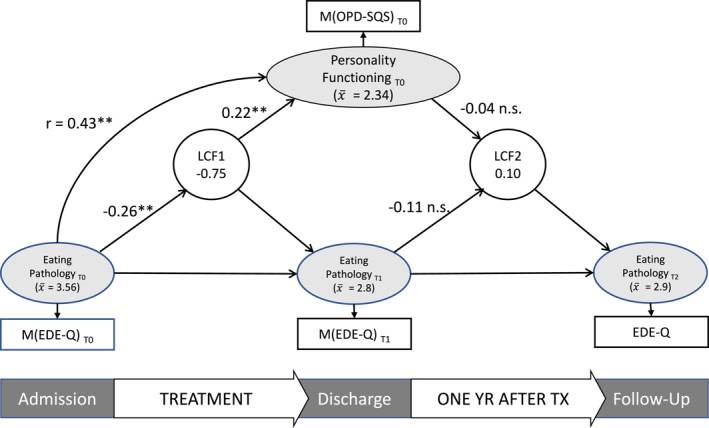
Model 1. Model with unstandardised coefficients; LCF = latent change score factor; T0 = admission; T1 = discharge; T2 = 1 year follow‐up; EDE‐Q = Eating Disorder Examination Questionnaire; OPD‐SQS = Operationalised Psychodynamic Diagnostics Structure Questionnaire, short version; YR = year; TX = treatment.

Figure 1 shows the results of the first LCSM, investigating the predictive effect of baseline PF on changes in ED pathology. Since this model was saturated, the model fit was perfect.

The level of PF and the severity of ED pathology at admission showed a highly significant, moderate positive correlation (*r* = 0.43, *p* < 0.001).

On average, ED symptom severity decreased (by ‐ 0.75 points) over the course of treatment and increased slightly during the follow‐up period (by 0.1 points). ED symptom severity at T0 was significantly associated with the change in ED pathology during treatment with more severe ED pathology at admission leading to a smaller change during treatment. However, ED symptom severity at T1 did not significantly influence changes in ED pathology during the follow‐up period. PF at admission significantly predicted changes in ED pathology during treatment: Patients with stronger impairments in PF showed smaller changes in ED pathology during treatment. PF at admission had no significant effect on changes in ED pathology during the 1‐year follow‐up period.

#### 3.1.3 Model 2

**FIGURE 2:**
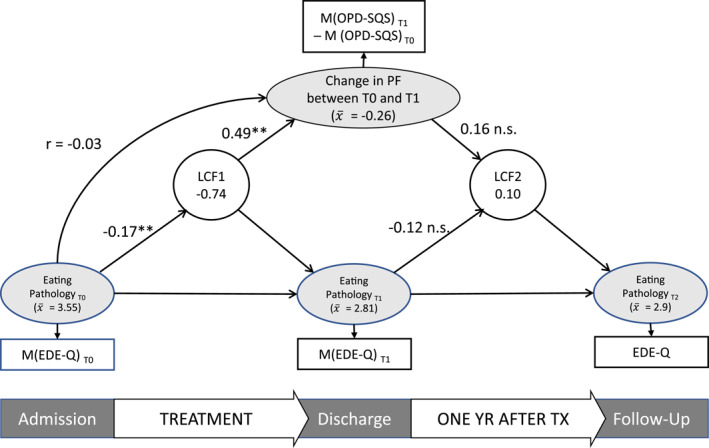
Model 2. Model with unstandardised coefficients; LCF1 = latent change score factor; T0 = admission; T1 = discharge; T2 = 1 year follow‐up; EDE‐Q = Eating Disorder Examination Questionnaire; OPD‐SQS = Operationalised Psychodynamic Diagnostics Structure Questionnaire, short version; PF = personality functioning; YR = year; TX = treatment.

The results for the second LCSM can be found in Figure 2. Here, we examined how changes in PF during treatment influenced changes in ED pathology during and after treatment. Since this model was saturated, the model fit was perfect.

On average, impairments in PF decreased during treatment by 0.26 points. As expected, changes in PF during treatment were not significantly associated with the severity of ED pathology at admission (*r* = −0.03, *p* = 0.569). Again, we found that more severe ED pathology at T0 led to smaller changes in ED pathology during treatment but that the severity of ED pathology at T1 did not have a significant influence on changes during the follow‐up period. Changes in PF during treatment showed a significant association with changes in ED pathology during treatment: Patients who showed more improvement in PF also showed more improvement in ED pathology. There was no significant influence of changes in PF during treatment on changes in ED pathology after discharge.

When comparing the models with standardised coefficients, the change in PF during treatment appears to be a better predictor of inter‐individual differences in changes in ED symptom severity during treatment (0.42) than the initial level of PF (0.23). When using the change in PF during treatment as a predictor, its influence on changes in ED symptom severity during treatment is greater than that of initial ED symptom severity, indicating the importance of addressing impairments in PF in hospital treatment of EDs. For the general model and models 1 and 2 with standardised coefficients see Supporting Information S1.

## 4 Discussion

We analysed the relationship between the level of overall impairment in PF and severity of ED psychopathology as well as treatment outcome in inpatients with EDs. PF refers to basic psychological capacities that encompass capabilities related to the organisation of the self and those related to interpersonal functioning (Ehrenthal et al. 2023). Higher levels of PF impairment at admission were associated with higher levels of baseline ED psychopathology in inpatients and day hospital patients with different ED diagnoses. The initial hypothesis was thus supported by the findings of the study. Furthermore, both a higher level of impairment in PF on admission and a reduction in PF impairment over the course of treatment significantly predicted a more positive treatment outcome at discharge, but not at follow‐up.

In line with our first hypothesis, we found a relationship between the level of PF and the severity of eating pathology at baseline. Such a relationship was also described by Rohde et al. (2023), although the association between PF and eating psychopathology in their study was weak when controlling for depression, anxiety and fatigue. The authors discuss two possible reasons for the weak relationship. The overall OPD‐SQ score may be closely related to the overall level of psychopathology, of which ED pathology is only a part of; on the other hand, there may be patients with similar levels of ED pathology but different levels of impairment in PF (Rohde et al. 2023). We did not control for further features of psychopathology and did not differentiate between diagnostic subgroups in this study. Therefore, the results are not directly comparable. However, since previous studies have shown a close association between difficulties in affect regulation, insecure attachment and a poor understanding of mental states, which are aspects of PF, and eating disordered behaviour (Caglar‐Nazali et al. 2014; Lavender et al. 2015), we would expect that greater impairments in PF will be associated with more disturbances in eating behaviour. In support of this, a population‐based study indicated that PF fully mediated the effect of attachment anxiety on ED symptomatology, further highlighting the significant role of PF in EDs (Klein et al. 2022).

The finding of an association between PF and eating pathology is also consistent with a study by Biberdzic, Tang, and Tan (2021) who studied a sample of 245 undergraduate students and found that difficulties in self‐regulation were strongly related to disordered eating. The authors suggest that on a ‘deeper level’, ED pathology might reflect impairment in PF. On the other hand, it could also be argued that severe ED symptoms may have secondary effects on PF (e.g., severe underweight may affect the perception of affect and cognitive processes) and that we are dealing with a complex process of mutual interaction.

Regarding the second research question, the findings showed that the severity of PF impairment was related to the outcome of hospital treatment, that is the amount of change in ED symptoms as measured with the EDE‐Q total score from admission to discharge. This finding aligns with results of studies in other mental disorders (Bach et al. 2020) and also with a study conducted in patients with EDs which showed that in 84 patients with AN and BN who were treated for 5.3 months in a residential treatment programme, healthy PF was associated with better treatment outcomes (Muzi et al. 2021b).

Thirdly, a greater improvement in PF over the course of treatment was associated with a greater change in ED symptoms. The amount of PF change was even a better predictor of outcome than initial ED symptom severity. Despite previous assumptions that PF or ‘personality structure’ is relatively stable over time, studies on psychodynamic inpatient treatment have shown, that PF can be improved by intensive psychotherapy over a time period of a few weeks (Kraus et al. 2021; Leichsenring et al. 2019). It seems to be more easily influenced by psychotherapy compared to the personality ‘style’ (Bach et al. 2020). For example, the improvement may be due to a change in self‐regulation or relationship skills. However, we did not differentiate between the different dimensions of PF. Furthermore, it is important to note that the type of the analysis does not allow us to draw causal conclusions. It is also possible that an improvement in ED symptoms (e.g. weight gain) improves PF.

Finally, neither PF at admission, nor change in PF between admission and discharge, nor severity of ED symptoms at discharge were able to predict the change in ED symptoms over the follow‐up period. This means that other unknown factors are more important. These may include the type of outpatient treatment including its intensity, positive or negative life events or the amount of social support. The finding is somewhat surprising as it is for example in contrast to a study which found a significant relationship between PF and symptom severity as well as disability 1 year post initial assessment (Weekers et al. 2024).

Strengths of the study encompass its multicenter design with a large sample and its external validity (sample of patients treated in the clinical routine). The analysis using a Latent Change Score Model (LCSM) allowed us to model the changes in several latent variables (personality functioning and ED pathology) over time and to quantify the interaction between the changes in PF and ED. The estimation of latent variables enables us to account for measurement error, resulting in estimates that are more reliable. The sample of patients studied can be considered representative of hospitalised adult patients with an ED in Germany. They were consecutively recruited in 19 university hospitals over a period of two years. It is important to mention that in Germany there is a tradition of intense inpatient psychotherapy programs. Patients are admitted not only for crisis intervention due to somatic complications or suicidal ideation, but for multimodal psychotherapeutic interventions when outpatient treatment was not sufficient or failed (Liebherz and Rabung 2014). The therapeutic focus is on symptom change (weight restauration, normalisation of eating patterns) as well as on an improvement in mental problem areas like affect regulation, interpersonal difficulties or self‐worth (Zeeck et al. 2006 2009).

However, the study also has several limitations. It only included patients admitted for hospital treatment in Germany with its programs that have a strong psychotherapeutic focus, therefore the findings cannot be generalised to outpatient samples and patients treated in other countries and types of programs. There may be variations between treatment centres in terms of the treatment programme and specialisation. We did not differentiate diagnostic subgroups of EDs (although differences in PF between different ED types have been shown in previous studies) and further aspects of comorbidity (level of depression or anxiety). We decided against controlling the models for anxiety and depression since this might reduce external validity, as many patients with ED also exhibit symptoms of these disorders. We did not aim to investigate the specificity of the influence of PF on ED compared with other disorders, such as anxiety and depression, but rather aimed for high field validity to inform inpatient treatment decisions. Furthermore, increasing the complexity of the model could jeopardise its stability of estimation. PF was assessed by a self‐report instrument. There may be patients with severe impairment in PF who do not score high on the OPD‐SQS as it requires an accurate self‐perception (which is part of PF). However, studies indicate a relatively high agreement between questionnaires and clinical interviews in assessing PF according to OPD (Benecke et al. 2009; Dinger et al. 2014). A further limitation of the present study lies in the predominant use of self‐report measures. While this method allows for standardised data collection with a good level of reliability and validity and the use of the participant's self‐insight, it may also lead to an overestimation of the associations between variables, as the extent of shared method variance is not controllable.

In sum, the study underlines the importance of a dimensional assessment of personality impairment, which was found to be associated with greater reliability and diagnostic stability than its categorial counterpart (Chmielewski et al. 2015).

## 5 Conclusion

The level of PF should be assessed at the beginning of treatment and taken into account when planning psychotherapeutic treatment for patients with an ED. Impairment in PF can be considered a non‐specific indicator of risk, as it is related both to treatment response and outcome and allows consideration of impairment in personality functioning even in the absence of a categorical diagnosis of a personality disorder. Furthermore, the findings suggest that patients may benefit from a psychotherapeutic treatment that focuses not only on ED pathology but also on areas of impairment in PF. Further studies should test our suggestion that changes in PF affect improvement in ED pathology (including the sequence of change), and address the question of whether tailoring interventions to the level of PF improves outcome. In conclusion, PF is a relevant variable that can inform clinical management and treatment planning in EDs.

## Ethics Statement

The original study was reviewed and approved by the Ethics Committee of the Medical Faculty of the Ruhr‐University Bochum on October 17, 2018 (ID: 18–6388) as well as all ethics committees of the recruiting centres including the local ethics committee of the University Hospital Freiburg (No 545/18). The patients/participants provided their written informed consent to participate in the study.

## Supporting information

Supporting Information S1

## Data Availability

Data are available upon reasonable request from the authors.
