# Supplementary material for: Personality Functioning in Inpatients With Eating Disorders: Association With Symptom Severity and Treatment Outcome
Source: Eur Eat Disord Rev. 2025 Feb 17;33(4):776–86. doi: 10.1002/erv.3183 (PMC12171681; doi:10.1002/erv.3183)
Supplement: Supplementary file 1 — Supporting Information S1 [file ERV-33-776-s001.pptx]

## Slide 1
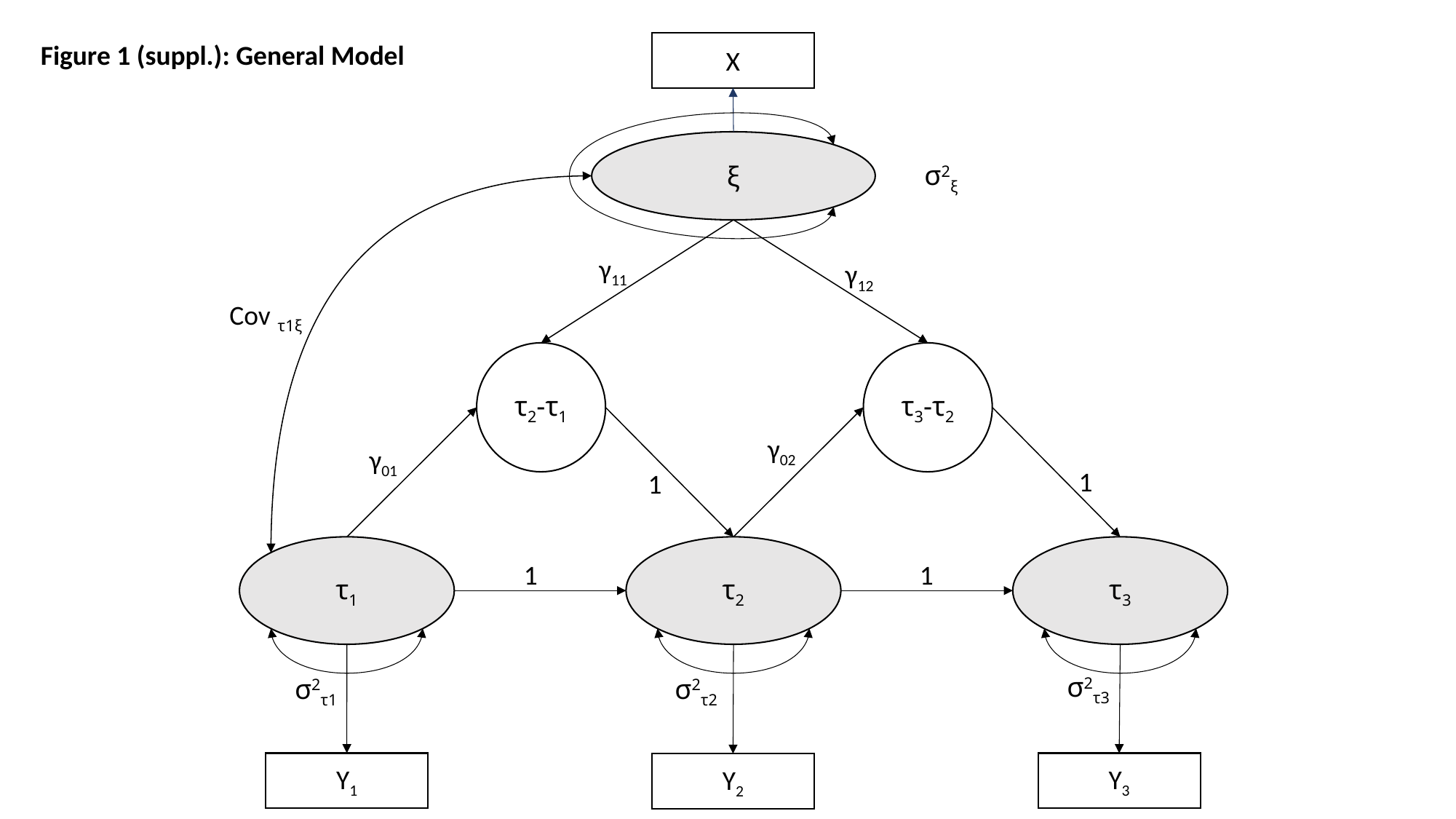

Figure 1 (suppl.): General Model
X
ξ
σ2ξ
γ11
γ12
Cov τ1ξ
τ2-τ1
τ3-τ2
γ02
γ01
1
1
τ2
τ3
τ1
1
1
σ2τ3
σ2τ1
σ2τ2
Y3
Y1
Y2

## Slide 2
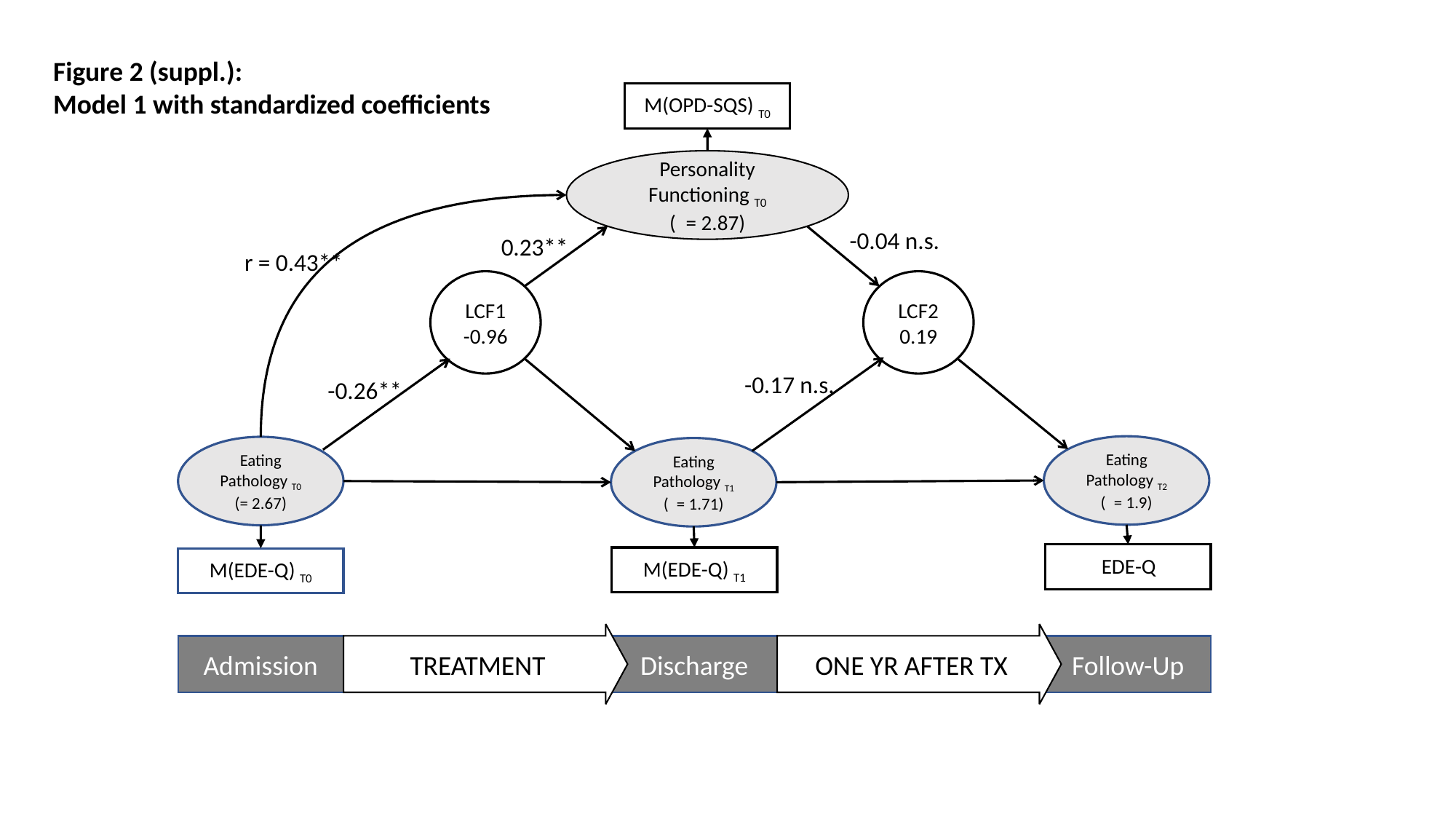

Figure 2 (suppl.):
Model 1 with standardized coefficients
M(OPD-SQS) T0
-0.04 n.s.
0.23**
r = 0.43**
LCF1
-0.96
LCF2
0.19
-0.17 n.s.
-0.26**
M(EDE-Q) T2
M(EDE-Q) T1
M(EDE-Q) T0
TREATMENT
ONE YR AFTER TX
Follow-Up
Admission
Discharge

## Slide 3
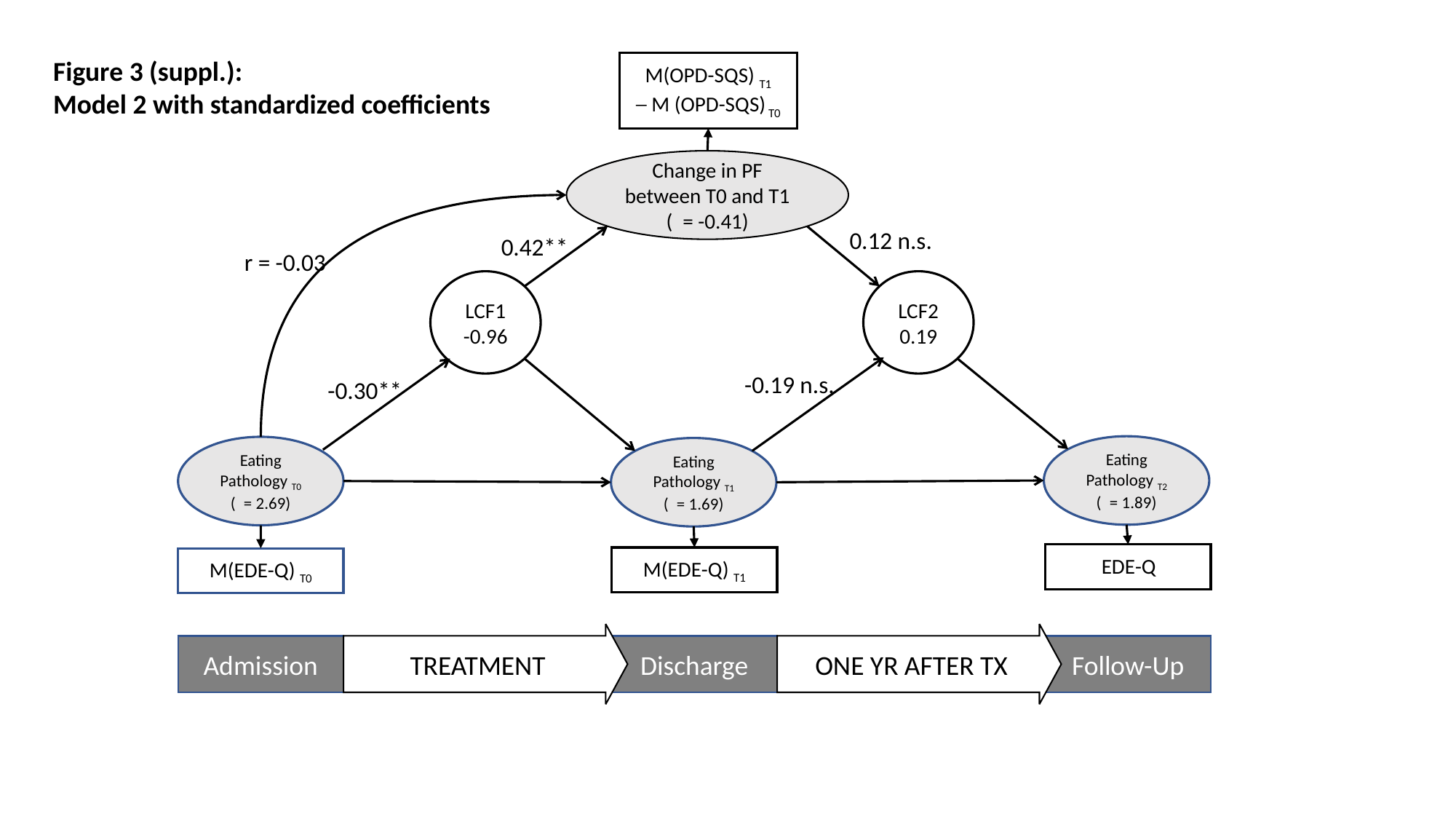

Figure 3 (suppl.):
Model 2 with standardized coefficients
M(OPD-SQS) T1
─ M (OPD-SQS) T0
0.12 n.s.
0.42**
r = -0.03
LCF1
-0.96
LCF2
0.19
-0.19 n.s.
-0.30**
M(EDE-Q) T2
M(EDE-Q) T1
M(EDE-Q) T0
TREATMENT
ONE YR AFTER TX
Follow-Up
Admission
Discharge
